# The frequency of rare and monogenic diseases in pediatric organ transplant recipients in Italy

**DOI:** 10.1186/s13023-021-02013-x

**Published:** 2021-09-04

**Authors:** Tiziana Vaisitti, Daniela Peritore, Paola Magistroni, Andrea Ricci, Letizia Lombardini, Enrico Gringeri, Silvia Catalano, Marco Spada, Marco Sciveres, Angelo Di Giorgio, Giuseppe Limongelli, Marisa Varrenti, Gino Gerosa, Amedeo Terzi, Carlo Pace Napoleone, Antonio Amodeo, Luca Ragni, Luca dello Strologo, Elisa Benetti, Iris Fontana, Sara Testa, Licia Peruzzi, Adele Mitrotti, Serena Abbate, Giorgia Comai, Eliana Gotti, Marco Schiavon, Massimo Boffini, Daniele De Angelis, Alessandro Bertani, Domenico Pinelli, Massimo Torre, Camilla Poggi, Silvia Deaglio, Massimo Cardillo, Antonio Amoroso, Italian Pediatric Transplant Centers, Enrico Gringeri, Silvia Catalano, Marco Spada, Marco Sciveres, Angelo Di Giorgio, Giuseppe Limongelli, Marisa Varrenti, Gino Gerosa, Amedeo Terzi, Carlo Pace Napoleone, Antonio Amodeo, Luca Ragni, Luca dello Strologo, Elisa Benetti, Iris Fontana, Sara Testa, Licia Peruzzi, Adele Mitrotti, Abbate Serena, Comai Giorgia, Eliana Gotti, Marco Schiavon, Massimo Boffini, Daniele De Angelis, Alessandro Bertani, Domenico Pinelli, Massimo Torre, Camilla Poggi, Silvia Deaglio, Massimo Cardillo, Antonio Amoroso

**Affiliations:** 1 Department of Medical Sciences, University of Torino, Via Nizza 52, 10126 Torino, Italy; 2 National Transplant Center, Istituto Superiore Di Sanità, Roma, Italy; 3 Immunogenetics and Transplant Biology, Azienda Ospedaliera Universitaria Città Della Salute E Della Scienza Di Torino, Torino, Italy; 4 Hepatobiliary Surgery and Liver Transplantation Unit, Padova University Hospital, Padova, Italy; 5 General Surgery 2U - Liver Transplant Center, Azienda Ospedaliera Universitaria Città Della Salute E Della Scienza Di Torino, University of Turin, Torino, Italy; 6 Divison of Hepatobiliopancreatic Surgery, Liver and Kidney Transplantation, Research Unit of Clinical Hepatogastroenterology and Transplantation, Bambino Gesù Children’s Hospital, IRCCS, Rome, Italy; 7 Department of Pediatrics, Ismett, Palermo, Italy; 8 Paediatric Hepatology, Gastroenterology and Transplantation, Papa Giovanni XXIII Hospital, Bergamo, Italy; 9 Center for Coordination on rare diseases – Regione Campania, Cardiovascular Rare and Genetic Diseases Unit, Department of Cardiology, Monaldi Hospital, AORN Dei Colli,, Naples, Italy; 10 DeGasperis CardioCenter, Niguarda Great Metropolitan Hospital, Milan, Italy; 11 Heart Transplantation Unit, Cardio-Thoraco-Vascular Sciences and Public Health Department, University Padova Hospital, Padova, Italy; 12 UOS Transplantation Surgery, Asst Papa Giovanni XXIII Hospital, Bergamo, Italy; 13 Pediatric Cardiac Surgery and Congenital Cardiopathies Unit, Regina Margherita Children’s Hospital, Azienda Ospedaliera Città Della Salute E Della Scienza Di Torino, Turin, Italy; 14 Bambino Gesù Children’s Hospital, IRCCS, Rome, Italy; 15 Paediatric Cardiology and ACHD Unit, S. Orsola, Malpighi Hospital, Bologna, Italy; 16 Renal Transplant Unit. Bambino Gesù Children’s Research Hospital IRCCS, Rome, Italy; 17 Pediatric Nephrology, Dialysis and Transplant Unit, Department of Women’s and Children’s Health, Padua University Hospital, Padua, Italy; 18 Azienda Ospedaliera Universitaria San Martino, Genoa, Italy; 19 Pediatric Nephrology, Dialysis and Transplant Unit, Fondazione IRCCS Ca’ Granda-Ospedale Maggiore Policlinico, Milan, Italy; 20 Pediatric Nephrology Unit, Regina Margherita Children’s Hospital, Azienda Ospedaliera Universitaria Città Della Salute E Della Scienza Di Torino, Turin, Italy; 21 Azienda Ospedaliera, Universitaria Policlinico Di Bari, Bari, Italy; 22 Department of Pediatrics, Ismett, Palermo, Italy; 23 Nephrology, Dialysis and Kidney Transplant Unit, IRCCS Azienda Ospedaliero-Universitaria Di Bologna, Bologna, Italy; 24 Unit of Nephrology, Azienda Socio Sanitaria Territoriale Papa Giovanni XXIII, Bergamo, Italy; 25 Thoracic Surgery and Lung Transplant Unit, Department of Cardiac, Thoracic, Vascular Sciences and Public Health, University Hospital of Padua, Padua, Italy; 26 Heart and Lung Transplant Center, Cardiac Surgery Division, Surgical Sciences Department, University of Torino, Torino, Italy; 27 Bambino Gesù Children’s Hospital, IRCCS, Rome, Italy; 28 Division of Thoracic Surgery and Lung Transplantation, Department for the Treatment and Study of Cardiothoracic Diseases and Cardiothoracic Transplantation, IRCCS-ISMETT, Palermo, Italy; 29 Department of Organ Failure and Transplantation, ASST Giovanni XXIII, Bergamo, Italy; 30 Ospedale Maggiore Policlinico, Milan, Italy; 31 Department of Thoracic Surgery, Policlinico Umberto I Hospital, University of Rome Sapienza, Rome, Italy

**Keywords:** Rare diseases, Monogenic diseases, Organ transplantation, Transplant outcome

## Abstract

**Background:**

Rare diseases are chronic and life-threatening disorders affecting < 1 person every 2,000. For most of them, clinical symptoms and signs can be observed at birth or childhood. Approximately 80% of all rare diseases have a genetic background and most of them are monogenic conditions. In addition, while the majority of these diseases is still incurable, early diagnosis and specific treatment can improve patients’ quality of life. Transplantation is among the therapeutic options and represents the definitive treatment for end-stage organ failure, both in children and adults. The aim of this paper was to analyze, in a large cohort of Italian patients, the main rare genetic diseases that led to organ transplantation, specifically pointing the attention on the pediatric cohort.

**Results:**

To the purpose of our analysis, we considered heart, lung, liver and kidney transplants included in the Transplant Registry (TR) of the Italian National Transplantation Center in the 2002–2019 timeframe. Overall, 49,404 recipients were enrolled in the cohort, 5.1% of whom in the pediatric age. For 40,909 (82.8%) transplant recipients, a disease diagnosis was available, of which 38,615 in the adult cohort, while 8,495 patients (17.2%) were undiagnosed. There were 128 disease categories, and of these, 117 were listed in the main rare disease databases. In the pediatric cohort, 2,294 (5.6%) patients had a disease diagnosis: of the 2,126 (92.7%) patients affected by a rare disease, 1,402 (61.1%) presented with a monogenic condition. As expected, the frequencies of pathologies leading to organ failure were different between the pediatric and the adult cohort. Moreover, the pediatric group was characterized, compared to the adult one, by an overall better survival of the graft at ten years after transplant, with the only exception of lung transplants. When comparing survival considering rare vs non-rare diseases or rare and monogenic vs rare non-monogenic conditions, no differences were highlighted for kidney and lung transplants, while rare diseases had a better survival in liver as opposed to heart transplants.

**Conclusions:**

This work represents the first national survey analyzing the main genetic causes and frequencies of rare and/or monogenic diseases leading to organ failure and requiring transplantation both in adults and children.

**Supplementary Information:**

The online version contains supplementary material available at 10.1186/s13023-021-02013-x.

## Background

Rare diseases are chronic and debilitating disorders affecting a small number of people compared to the general population, with small differences in definition. In the USA, a disease is considered to be rare when it affects < 200,000 people in the country, while in Europe the frequency is < 1 in 2,000 people (1, 2). In addition, there are geographical issues, as diseases that are generally rare can be frequent in a specific population. For example, the congenital nephrotic syndrome of the Finnish type is generally a rare disease, with the exception of Finland, where it occurs more frequently than in other parts of the world (3). Although each rare disease affects a small number of patients, globally considered they result in roughly 400 million patients.

Approximately 80% of all rare diseases are genetic in origin and most of them display a family distribution compatible with a monogenic origin (4, 5), even though in a proportion of disorders the causative gene remains elusive (5, 6). In this context, the recent evolution and broader application of sequencing technologies have revealed the genetic causes of novel rare disease and led to the identification of new variants responsible for previously defined disorders (7).

Rare diseases are often progressive conditions and for most of them clinical symptoms and signs can be observed at birth or childhood, being responsible for 35% of deaths in the first year of life and a significant cause of pediatric hospital admissions (8–10). Moreover, in a considerable percentage of cases, they are severe multisystem disorders displaying a range of phenotypes with consequent diagnostic and patient management challenges. Notably, the great majority (95%) of the 7,000–8,000 estimated rare diseases still lack FDA/EMEA approved therapies (11), even though symptomatic treatment and medical care can improve patients’ quality of life and extend life expectancy.

Transplantation is one of the options and represents the definitive treatment for end-stage organ failure, both in children and adults. The advent of new immunosuppressive drugs and the improvement of surgical techniques have contributed to its widespread diffusion, which in turn has led to an expansion of medical indications and an increased organ need (12, 13).

This heightened demand, together with a non-proportional raise in organs availability, impose the ethical need that “no organ can be used for futile transplants or burdened by a poor prognosis” (14, 15). In 2019, a total of 34,285 transplants were performed in Europe (21,235 kidney; 7,900 liver; 2,269 heart; 2,136 lung; 710 pancreas and 35 small bowel), which is however a small fraction when compared to the number of waiting listed patients in the same period (109,739 patients in total: 79,513 kidney; 16,007 liver; 6,940 heart; 4,883 lung; 2,313 pancreas and 83 small bowel) (16).

An open point in transplantation is the impact of rare diseases, specifically the ones with a genetic origin, since a significant percentage of end-stage organ failures are caused by monogenic pathologies (17, 18). Even though a wide variety of rare diseases benefit from solid organ transplantation, few reports address this topic from the genetic point of view.

The main aim of this paper is to analyze, in a large cohort of Italian patients, which are the main genetic diseases that lead to organ transplantation. Specifically, we aimed to shed light on pediatric transplantation, evaluating which among these pathologies lead more frequently to transplantation, the frequencies of rare and monogenic diseases and the organs involved. Finally, we analyzed the transplant outcome comparing patients with rare diseases—distinguishing even the most frequent monogenic ones – with those affected by rare non-monogenic diseases.

## Results

### Cohort selection

In the January 2002—December 2019 timeframe, the Transplant Registry (TR) of the National Transplantation Center database recorded 59,941 solid organ transplants, consecutively performed in Italy, of which 56,907 from cadaveric donors.

To the purpose of this study, due to low numbers, single pancreas (n = 1,146) and small bowel (n = 55) transplants were excluded from analysis, while re-transplanted patients were considered only once. Moreover, the cohort included recipients who underwent combined organ transplantation, both same organ (e.g., bi-pulmonary or bi-renal) and multi-organs (4,372 in total; Additional file 1: Table 1). Overall, our analysis, included kidney (n = 25,407), liver (n = 17,207), heart (n = 4,868), and lung (1,922) transplants, resulting in an enrolled cohort of 49,404 recipients (Table 1). In 40,909 (82.8%) transplants, a definite diagnosis of disease leading to organ failure was available, of which 38,615 in the adult cohort and 2,294 in the pediatric one, while 8,495 patients (17.2%) were present in the TR without a diagnosis. Transplants were performed in 47 Italian Transplant Centers, of which 34 involved in pediatric activities (Additional file 1: Table 2).

**Table 1:** Transplanted organ category and main features of the cohort included in the study

	Transplanted organ				
	Kidney	Liver	Heart	Lung	Total
N. of transplants	25,407	17,207	4,868	1,922	49,404
Without diagnosis of the original disease	5,967	334	1,611	583	8,495
With diagnosis	19,440	16,873	3,257	1,339	40,909
Adults	18,553	15,836	2,981	1,245	38,615
Pediatrics	887	1,037	276	94	2,294
N. of pediatric disease categories	66	39	14	9	128
N. of pediatric diseases included in the rare disease catalogs	61	35	13	8	117
N. of pediatric patients diagnosed with a rare disease	840	920	273	93	2,126
N. of pediatric patients diagnosed with a monogenic disease	411	686	223	82	1,402

We then focused the attention on the pediatric cohort, defined as patients who were less than 18 years old when enrolled in the waiting list, and included in the TR with a disease diagnosis (n = 2,294). The mean age at the time of enrollment in the TR was 7.15 years, IQR [1–13 years], with differences when considering the type of organ (heart: 7.84; liver: 4.06; lung: 13.04; kidney: 9.86), while the mean age at the time of transplantation was 7.99 years, IQR [1–14].

To understand the impact of rare monogenic pathologies in this cohort, we matched the diseases categories entered in the TR with those listed in the main rare diseases databases. Based on TR data entries, overall, there were 128 disease categories: 66 for kidney, 39 for liver, 14 for heart and 9 for lung (Table 1). Of them, 117 were listed in the main rare disease databases. Within the pediatric cohort, 2,126 out of 2,294 (92.6%) presented with a rare disease (840 kidney; 920 liver; 273 heart and 93 lung) of which 1,402 affected by a monogenic disease (411 kidney; 686 liver; 223 heart and 82 lung; Table 1).

Overall, the median follow-up from the transplant date was similar for the organs considered in the study: 6.73 years, IQR [2.48–10.57] for kidney; 5.39 years, IQR [1.23–8.87] for liver; 6.39 years, IQR [1.32–10.74] for heart; 3.6 years, IQR [0.39–5.62] for lung.

### Transplants in the Italian pediatric and adult cohorts: state of the art

We then concentrated on patients with a clear diagnosis, looking at the distribution of transplants in the Italian cohort comparing the pediatric and adult groups, and taking in consideration i) transplanted organs, ii) main disease macro-categories that led to organ failure and iii) graft survival at 10 years after transplant. For the sake of clarity, this paragraph was divided in subparagraphs focused on the different transplanted organs.

#### Heart

For 3,257 heart transplanted patients (66.9% of all heart transplants) a diagnosis was recorded in the TR; of them 276 were children (8.5%) and 2,981 adults (91.5%). While cardiomyopathies represented the first cause of disease both in children (63.8%) and adults (53.6%), coronary heart disease and valve heart disease were prevalent in adults (31.5% vs 1.1% and 5.4% vs 1.8%, respectively). On the other hand, children were significantly more affected by congenital heart disease than adults (30.4% vs 2.8%). It is also noteworthy that in the adult group, a significant proportion of patients were affected by other cardio-circulatory diseases (5.8%; Table 2).

**Table 2:** Heart transplant cohort and main disease categories leading to transplantation

Disease macro-category	Pediatrics (n. 276)	Adults (n. 2,981)	Total (n. 3,257)
	n. (%)	n. (%)	n. (%)
Cardiomyopathies	176 (63.8)	1,597 (53.6)	1,773 (54.4)
Congenital heart disease	84 (30.4)	82 (2.8)	166 (5.1)
Valve heart disease	5 (1.8)	161 (5.4)	166 (5.1)
Coronary disease	3 (1.1)	939 (31.5)	942 (28.9)
Primary lung hypertension	0 (0.0)	3 (0.1)	3 (0.1)
Other congenital pathologies	3 (1.1)	7 (0.2)	10 (0.3)
Other cardio-circulatory diseases	5 (1.8)	192 (6.4)	197 (6.0)

**Fig. 1:**
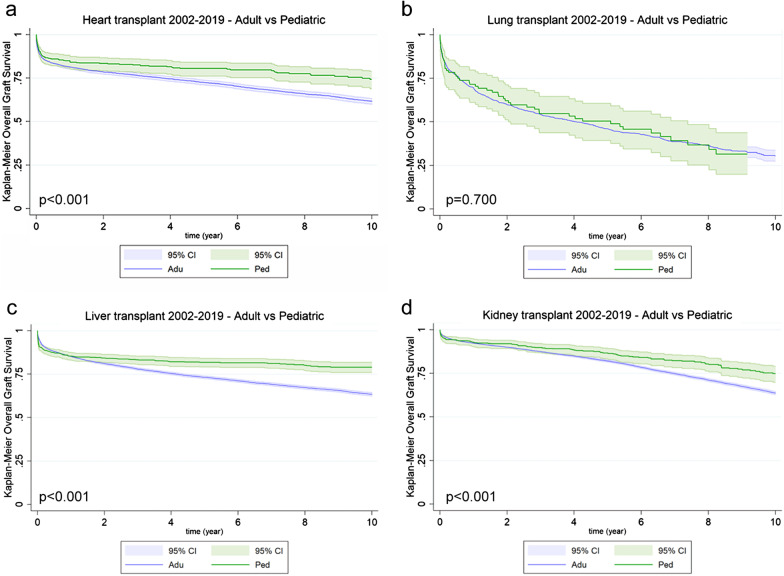
Overall graft survival of transplanted patients in the 2002–2019 timeframe. Kaplan–Meier curves comparing adult (purple line) vs. pediatric (green line) transplanted cohort. Heart (*p* < 0.001; 4248 adults vs 403 children; **a)**; Lung (p = 0.700; 1445 adults vs 94 children; **b)**; Liver (*p* < 0.001; 14,215 adults vs 967 children; **c)** and Kidney (*p* < 0.001; 17,588 adults vs 817 children; **d)**

When looking at graft survival, children had a better outcome than adults (*p* < 0.001, Fig. 1a).

#### Lung

Of the 1,922 lung transplants, 1,339 (69.7%) had a detailed disease diagnosis. The analysis therefore focused on 1,245 adults (93%) and 94 pediatric cases (7%; Table 1). In children, in line with recent published data (19), the most frequent cause of organ failure was cystic fibrosis (79.8%), followed by primary pulmonary hypertension (7.4%), congenital disorders and other pneumopathies (4.3% each), which together accounted for 95.8% of cases. On the contrary, in adult patients, idiopathic pulmonary fibrosis was the most frequent cause of disease (35.9%), followed by cystic fibrosis (28.4%), emphysema/chronic obstructive pulmonary disease (18.4%) and other pneumopathies (11.6%). These 4 categories explained approximately 95% of cases (Table 3).

**Table 3:** Lung transplant cohort and main disease categories leading to transplantation

Disease macro-category	Pediatrics (n. 94)	Adults (n. 1,245)	Total (n. 1,339)
	n. (%)	n. (%)	n. (%)
Cystic fibrosis	75 (79.8)	354 (28.4)	429 (32.0)
Primary pulmonary hypertension	7 (7.4)	51 (4.1)	58 (4.3)
Other pneumopathies	6 (6.4)	148 (11.9)	154 (11.5)
Congenital disorders	4 (4.3)	1 (0.1)	5 (0.4)
Emphysema/Chronic obstructive pulmonary disease	1 (1.1)	229 (18.4)	230 (17.2)
Idiopathic pulmonary fibrosis	1 (1.1)	447 (35.9)	448 (33.5)
Congenital heart disease	0 (0.0)	4 (0.3)	4 (0.3)
Alpha-1 antitripsin deficiency	0 (0.0)	11 (0.9)	11 (0.8)

The success of lung transplantation was similar in pediatric and adult patients, with no significant differences at 10 years (Fig. 1b), even when excluding cystic fibrosis from the cohort (data not shown), in line with previously reported data (20).

#### Liver

Out of the 17,207 liver recipients, 1,077 were performed in children (6.3%) and 16,873 patients (98.1% of cases) were registered in the TR with a diagnosis. There were 462 different disease definitions, which could be grouped into 9 diagnostic macro-categories, as shown in Table 4. Specifically, in the pediatric cohort, the most represented diseases were atresia of the biliary tract (41.8%), other hepatopathies (17.3%), metabolic diseases (12.2%), cholestatic cirrhosis (11.7%), and neoplasia (8%), accounting for 94% of all cases. On the contrary, in adults, the most frequent diagnoses were non-cholestatic cirrhosis (37.6%) and liver neoplasia (28.4%), followed by alcoholic cirrhosis (10.6%), cholestatic cirrhosis and cholestatic diseases (5.4%), other liver diseases (5.5%), accounting for 90% of all cases (Table 4).

**Table 4:** Liver transplant cohort and main disease categories leading to transplantation

Disease macro-category	Pediatrics (n. 1,037)	Adults (n. 15,836)	Total (n. 16,873)
	n. (%)	n. (%)	n. (%)
Hepatopathies from atresia of the biliary tract	433 (41.8)	32 (0.2)	465 (2.8)
Metabolic diseases	126 (12.2)	350 (2.2)	476 (2.8)
Cholestatic cirrhosis and other cholestatic liver diseases	121 (11.7)	857 (5.4)	978 (5.8)
Neoplasia	83 (8.0)	4,505 (28.4)	4,588 (27.2)
Acute liver necrosis	44 (4.2)	404 (2.6)	448 (2.7)
Other non-cholestatic cirrhosis	43 (4.1)	5,950 (37.6)	5,993 (35.5)
HBV/HCV related cirrhosis	5 (0.5)	1,116 (7.0)	1,121 (6.6)
Alcoholic cirrhosis	0 (0.0)	1,686 (10.6)	1,686 (10.0)
Other hepatopathies	182 (17.5)	936 (5.9)	1,118 (6.6)

When comparing the probability of graft success between the pediatric and adult cohort, the former presented an overall better survival than adults (*p* < 0.001; Fig. 1c).

#### Kidney

Lastly, we analysed kidney transplants with 25,407 patients included in the TR. 19,440 recipients were registered with a disease diagnosis (76.5%), of which the great majority (95.4%) were adults with only 887 pediatric patients (4.6%; Table 1). There were 568 different disease definitions, which could be grouped into 13 macro-categories (Table 5). In pediatric transplants, congenital familial nephro- and uropathies were the most represented pathologies (75.5% vs 2% in adults), followed by glomerular (10%) and cystic (4.8%) nephropathies. On the other hand, the most frequent pathologies in adult kidney transplants were glomerular nephropathies (40.1%), followed by cystic nephropathies (18.8%), other renal disorders (11%), hypertensive nephrosclerosis (8.7%), diabetic nephropathy (7.6%) and tubular and interstitial nephropathies (7.1%), the latter 3 macro-categories being almost absent in the pediatric cohort.

**Table 5:** Kidney transplant cohort and main disease categories leading to transplantation

Disease macro-category	Pediatrics (n. 887)	Adults (n. 18,553)	Total (n. 19,440)
	n. (%)	n. (%)	n. (%)
Congenital familial rare and metabolic based nephro- and uro-pathies	670 (75.5)	378 (2.0)	721 (3.7)
Glomerular nephropathies	89 (10.0)	7,438 (40.1)	7,527 (38.7)
Cystic nephropathies	43 (4.8)	3,493 (18.8)	3,536 (18.2)
Acute renal insufficiency	13 (1.5)	43 (0.2)	56 (0.3)
Other familial nephropathies	12 (1.4)	227 (1.2)	239 (1.2)
Different nephro-vasculopathic and nephrosclerosis syndromes	7 (0.8)	389 (2.1)	396 (2.0)
Kidney neoplasia	2 (0.2)	53 (0.3)	55 (0.3)
Hypertensive nephrosclerosis	1 (0.2)	1,610 (8.7)	1,611 (8.3)
Tubular and interstitial nephropathies	0 (0.0)	1,326 (7.1)	1,326 (6.8)
Diabetic nephropathy	0 (0.0)	1,409 (7.6)	1,409 (7.2)
Non-cholestatic cirrhosis	0 (0.0)	2 (0.0)	2 (0.0)
Other hepatopathies	3 (0.3)	1 (0.0)	4 (0.0)
Other kidney disorders	47 (5.3)	2,184 (11.8)	2,558 (13.2)

Kaplan–Meier curves of the overall graft survival referred to kidney and comparing the pediatric and adult cohorts, showed that the former one was characterized by a better outcome (*p* < 0.001; Fig. 1d).

### Rare and monogenic diseases frequencies in pediatric transplanted patients and their impact on grafts survival

In the last section of the work, we focused selectively on the pediatric cohort of transplanted patients, included in the TR with a clear diagnosis, with the aim of defining the frequencies of rare and/or monogenic diseases that were causative of organ failure, ultimately requiring organ transplantation. For the former group of pathologies, we firstly matched the main databases reporting these diseases to obtain a full list for each organ. Rare diseases are indicated with the Orphanet code. The diagnoses included in the TR were then classified according to disease categories in rare and rare monogenic diseases. As a last inquiry, we compared graft survival in patients affected by i) rare vs non-rare diseases, ii) rare monogenic vs rare non-monogenic diseases and finally iii) considering the most represented disease macro-categories for each organ.

13 out of 14 diseases categories responsible for heart transplantation in children were included in the rare disease catalogues, accounting for almost all pediatric patients with heart transplantation (273 out of 276, 98.9%), with 223 patients (82%) diagnosed with a pathology belonging to monogenic diseases. The only condition found in pediatric cases that is not considered a rare disease was dilated heart disease on ischemic basis (3 patients). However, it is not possible to exclude with certainty that even in these cases a rare/monogenic pathology was involved, possibly due to disorders of lipid metabolism. The most represented pathology was dilated cardiomyopathy (42.8% of cases), followed by congenital heart disease on a genetic basis (30.8%), hypertrophic cardiomyopathy (8.3%) and restrictive cardiomyopathy (7.6%). Alone, these 4 disease categories represented 89% of pediatric transplants (Table 6).

**Table 6:** Diagnostic categories in heart transplanted pediatric cohort and distribution between rare and monogenic diseases

Diagnosis	ORPHA code	Tx (n. 276)	Rare diseases (n. 273)	Monogenic diseases (n. 223)
			n. (% respect to total Tx)	n. (% respect to rare diseases)
Complication following organ transplant	306,644	1	1 (0.4)	
Dilated cardiomyopathy	217,604	118	118 (42.8)	113 (41.4)
Dilated cardiomyopathy on ischemic basis		3		
Ebstein malformation	1880	1	1 (0.4)	1 (0.4)
Familial isolated arrhythmogenic dysplasia of the right ventricle	217,656	1	1 (0.4)	1 (0.4)
Genetic heart disease	271,853	85	85 (30.8)	84 (31.1)
Hypertrophic cardiomyopathy	217,569	23	23 (8.3)	
Hypoplastic left heart	2248	2	2 (0.7)	2 (0.7)
Myocarditis	§	9	9 (3.3)	
Radiation-induced pathology	521,132	5	5 (1.8)	
Restrictive cardiomyopathy	217,632	21	21 (7.6)	21 (7.6)
Shone Complex	99,063	1	1 (0.4)	1 (0.4)
Severe aortic insufficiency	99,094	1	1 (0.4)	
Valvular heart disease	228,410	5	5 (1.8)	

Tx: transplants;

^§^: included in the NORD database ( https://rarediseases.org/rare-diseases/myocarditis/)

**Fig. 2:**
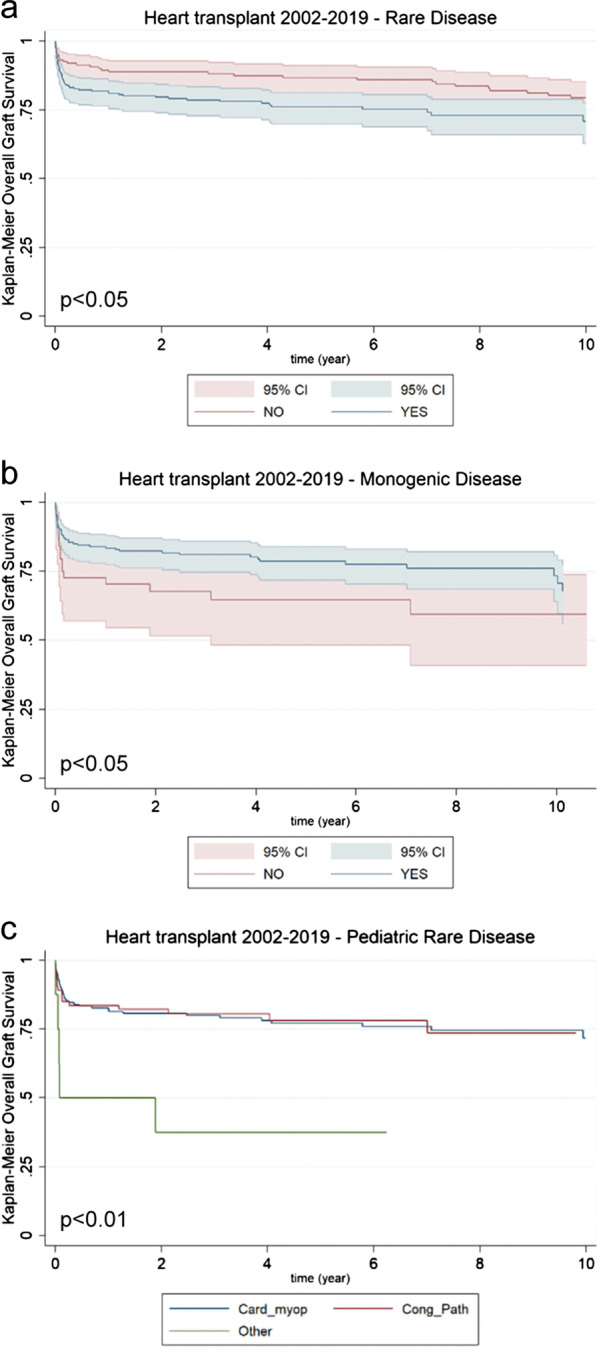
Survival of heart transplanted patients in the 2002–2019 timeframe. **a** Kaplan–Meier curves comparing patients affected by rare diseases (green line; n = 240) vs. patients affected by non-rare diseases (red line; n = 163) [*p* < 0.05]. **b** Kaplan–Meier curves comparing patients affected by monogenic diseases (green line; n = 196) vs. patients affected by rare non-monogenic diseases (red line; n = 44) [*p* < 0.05]. **c** Kaplan–Meier curves comparing patients affected by cardiomyopathies (Card_myop; blu line; n = 158) vs. congenital pathologies (Cong_Path; red line; n = 74) vs. other pathologies (Other; green line; n = 8) [*p* < 0.01]

If we distinguish pediatric transplants according to whether they have been performed because of a rare vs a non-rare disease, we noted that the probability of success was significantly better for transplants performed in children without rare diseases (*p* < 0.05; Fig. 2a). On the contrary, transplants performed because of a monogenic disease presented a significantly better chance of success (*p* < 0.05; Fig. 2b). Finally, when considering the outcome of the transplant taking into account the 2 most represented groups of cardiac diseases, congenital pathologies and cardiomyopathies, no significant differences were highlighted between them, even though they showed a significant better profile compared to other cardiac pathologies (*p* < 0.01; Fig. 2c).

When analysing in detail the 9 different definitions of disease found in lung transplanted patients and classifying them according to rare diseases, we noted that almost all patients (98.9%) were affected by rare diseases, of whom 86% of monogenic origin. Cystic fibrosis was the leading cause of lung failure, affecting 80.6% of the whole cohort. The second most frequent diagnosis was represented by pulmonary arterial hypertension, a rare disease of non-monogenic nature, counting 7.5% of patients.

(Table 7).

**Table 7:** Diagnostic categories in lung transplanted pediatric cohort and distribution between rare and monogenic diseases

Diagnosis	ORPHA code	Tx (n. 94)	Rare diseases (n. 93)	Monogenic diseases (n. 82)
			n. (% respect to total Tx)	n. (% respect to rare diseases)
Bronchiolitis obliterans	1303	2	2 (2.1)	
Chronic obstructive pulmonary disease		1		
Cystic fibrosis	586	75	75 (80.6)	75 (80.6)
Eisenmenger syndrome	97,214	4	4 (4.3)	4 (4.3)
Idiopathic pulmonary fibrosis	2032	1	1 (1.1)	1 (1.1)
Interstitial lung disease due to ABCA3 deficiency	440,402	1	1 (1.1)	1 (1.1)
Langerhans cell histiocytosis	389	1	1 (1.1)	1 (1.1)
Pulmonary arterial hypertension	182,090	7	7 (7.5)	
Rejection/Complication after organ transplantation	306,644	2	2 (2.1)	

Tx: transplants

**Fig. 3:**
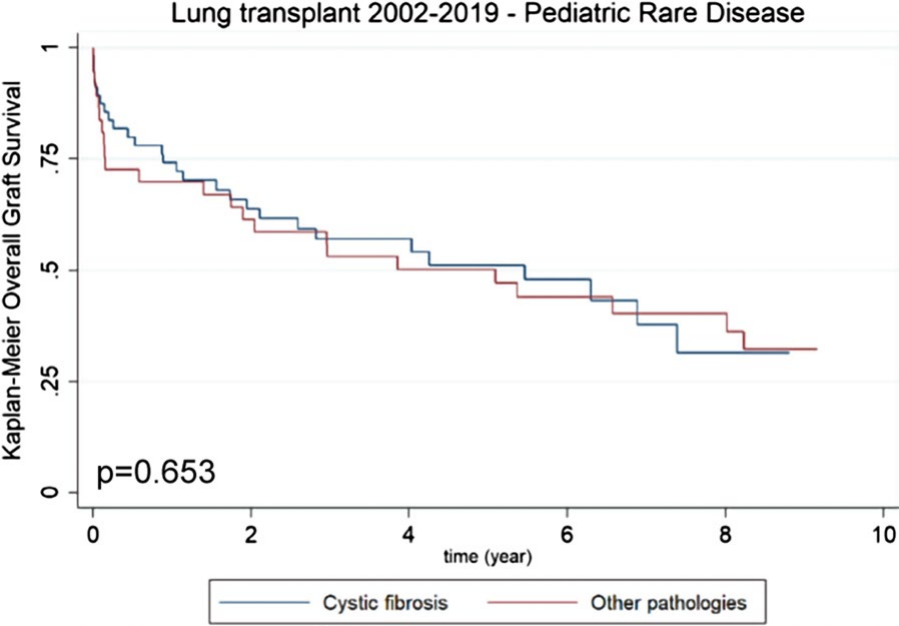
Survival of lung transplanted patients in the 2002–2019 timeframe. Kaplan–Meier curves comparing pediatric patients affected by cystic fibrosis (blue line; n = 56) vs. pediatric patients affected by other pathologies (red line; n = 37) [p = 0.653]

Since more than 50% of pediatric transplants were performed in recipients with cystic fibrosis, which is a rare and monogenic disease, further evaluations in relation to the outcome of pediatric transplants were strongly influenced by this single category. Moreover, due to an almost complete overlap between rare and monogenic diseases also in the remaining categories, we did not perform survival analyses. And indeed, the overall survival between cystic fibrosis diagnosed patients and all the other disease categories showed no differences (p = 0.653; Fig. 3), in line with previously reported data (19).

Regarding liver pediatric transplants, there were 39 different definitions of diseases available in the TR, with the great majority included in the catalogues of rare diseases (36 out of 39 disease categories, accounting for 90% of patients) and 17 out of 39 being of monogenic origin, including almost 71% of patients. Analyzing the distribution of these diagnoses, it was noted that the most frequent were congenital biliary disease/extrahepatic biliary atresia (41.1% of patients), cholestatic diseases (11.5%), metabolic diseases, including cystic fibrosis and primary hyperoxaluria (9.8%), Alagille syndrome (4.5%), and Wilson disease (1.7%), which all together constituted approximately 70% of all cases (Table 8).The distribution in this cohort is slightly a bit different from the ones previously reported by Fagiuoli and colleagues, where the dominant phenotype was represented by Alagille syndrome (21).

**Table 8:** Diagnostic categories in liver transplanted pediatric cohort and distribution between rare and monogenic diseases

Diagnosis	ORPHA code	Tx (n. 1,037)	Rare diseases (n. 904)	Monogenic diseases (n. 689)
			n. (% respect to total Tx)	n. (% respect to rare diseases)
Acute liver failure—Fulminant or subfulminant hepatic failure		53		
Alagille syndrome	52	47	47 (4.5)	47 (5.2)
Alpha-1 antitrypsin deficiency	60	11	11 (1.1)	11 (1.2)
Angiosarcoma (neoplasia)	263,413	1	1 (0.1)	
Budd-Chiari syndrome	131	5	5 (0.5)	5 (0.5)
Caroli disease	53,035	6	6 (0.6)	
Cholestatic disease—Primary biliary cirrhosis	186	17	17 (1.6)	
Cholestatic disease—Primary sclerosing cholangitis	171	42	42 (4.1)	
Cholestatic disease—Other		27		
Cholestatic disease—Secondary biliary cirrhosis	447,774	29	29 (2.9)	
Cirrhosis—Autoimmune	779	8	8 (0.8)	
Cirrhosis—Other		10		
Cirrhosis—Virus related		11		
Congenital biliary disease—Extrahepatic biliary atresia	498,345	426	426 (41.1)	426 (47.1)
Crigler-Najjar syndrome type 1	79,234	4	4 (0.4)	4 (0.4)
Cryptogenic cirrhosis		24		
Epithelioid hemangioendothelioma (neoplasia)	157,791	3	3 (0.3)	
Familial hypercholesterolemia	391,665	1	1 (0.1)	
Glycogen storage disease	79,201	6	6 (0.6)	6 (0.6)
GVHD	99,921	3	3 (0.3)	
Hemochromatosis	220,489	5	5 (0.5)	5 (0.5)
Hepatic trauma		1		
Hepatoblastoma (neoplasia)	449	55	55 (5.3)	
H epatocellular (neoplasia)	88,673	19	19 (1.8)	
Hepatocellular carcinoma—Fibrolamellar (neoplasia)	401,920	2	2 (0.2)	
Isolated Congenital hepatic fibrosis	485,426	19	19 (1.8)	19 (2.1)
Langherans cell histiocytosis	389	3	3 (0.3)	
Metabolic disease—Cystic fibrosis	586	17	17 (1.6)	17 (1.9)
Metabolic disease—Others	91,088	49	49 (4.7)	49 (5.4)
Metabolic disease—Primary hyperoxaluria	416	25	25 (2.4)	25 (2.8)
Metabolic disease—Tyrosinemia	882/28378/ 68,723	11	11 (1.1)	11 (1.2)
Methylmalonic acidemia with homocystinuria, type cblD	79,283	3	3 (0.3)	3 (0.3)
Neonatal intrahepatic cholestasis caused by citrin deficiency	247,598	1	1 (0.1)	1 (0.1)
Other neoplasia		7		
Other rare liver diseases	^#^	26	26 (2.5)	
Polycystic liver disease	2924	6	6 (0.6)	6 (0.6)
Progressive familial intrahepatic cholestasis	172	36	36 (3.5)	36 (4.0)
Wilson disease	905	18	18 (1.7)	18 (2.0)

Tx: transplants; GVHD: graft-versus-host disease; # several ORPHA codes

**Fig. 4:**
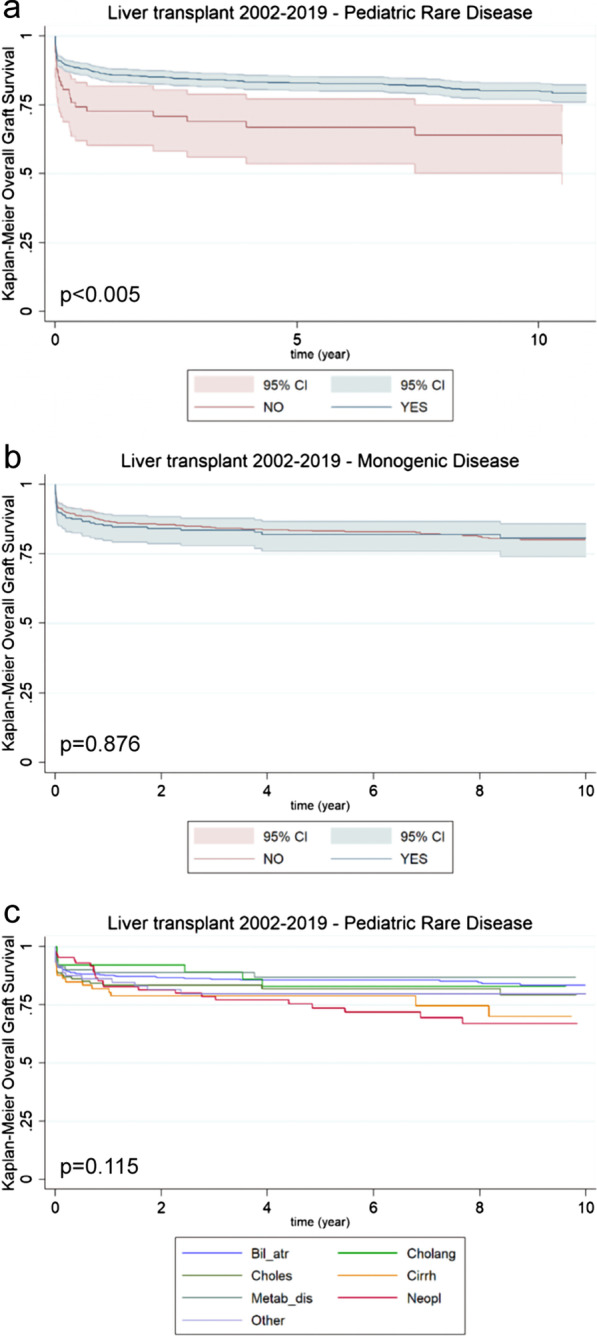
Survival of liver transplanted patients in the 2002–2019 timeframe. **a** Kaplan–Meier curves comparing pediatric patients affected by rare diseases (green line; n = 900) vs. pediatric patients affected by non-rare diseases (red line; n = 67) [*p* < 0.005]. **b** Kaplan–Meier curves comparing pediatric patients affected by monogenic diseases (green line; n = 227) vs. pediatric patients affected by rare non-monogenic diseases (red line; n = 673) [p = 0.876]. **d** Kaplan–Meier curves comparing pediatric patients affected by rare diseases stratified on the basis of their diagnosis [p = 0.11]: biliary atresia (Bil_atr; blue line; n = 420); cholestasis (Choles; light green line; n = 116); metabolic diseases (Metab_dis; grey line; n = 92); Cholangitis (Cholang; green line; n = 40); cirrhosis (Cirrh; yellow line; n = 73); neoplasia (Neopl; red line; n = 86); other diseases (Other; purple line; n = 73)

Pointing the attention on the graft survival of pediatric liver transplants, those performed in patients with rare diseases showed a significantly better outcome compared to patients with common diseases (*p* < 0.005; Fig. 4a). On the contrary, no differences were noted when dividing them based on the presence or absence of a monogenic disease (p = 0.87; Fig. 4b). When plotting graft survival according to the main diagnostic categories, globally considered, no statistically significant differences were highlighted (p = 0.11). However, a trend towards a better outcome could be noted for metabolic diseases, biliary atresia, and cholestatic diseases. On the contrary, transplants performed because of neoplasia and cirrhosis showed a worse overall survival (Fig. 4c).

Lastly, we looked at kidney transplants in the pediatric cohort. The list contained 66 different disease categories, most of which (n = 62) included in the catalogues of rare diseases. Overall, 94.7% of transplanted patients had a diagnosis of rare disease and half of these ones (46.3%) had a monogenic origin. The most represented diseases were renal or urinary tract malformation (36.9%), followed by focal segmental glomerulosclerosis (8.1%), nephronophthisis (8%), autosomal recessive polycystic kidney disease (4.8%), glomerulonephritis (4.3%), congenital nephrotic syndrome Finnish type (3.8%), Alport syndrome (3%) and hemolytic uremic syndrome (2.9%). It is worthy to note that monogenic diseases might be underestimated, as monogenic forms of renal or urinary tract malformation and glomerulonephritis are known (Table 9).

**Table 9:** Diagnostic categories in kidney transplanted pediatric cohort and distribution between rare and monogenic diseases

Diagnosis	ORPHA code	Tx (n. 887)	Rare diseases (n. 837)	Monogenic diseases (n. 413)
			n. (% in respect to total Tx)	n. (% respect to rare diseases)
Alport syndrome	63	27	27 (3.0)	27 (3.3)
Autosomal dominant tubulointerstitial kidney disease	34,149	3	3 (0.3)	3 (0.4)
Autosomal dominant tubulointerstitial kidney disease due to HNF1B	93,111	1	1 (0.1)	1 (0.1)
Autosomal dominant tubulointerstitial kidney disease due to UMOD mutation	88,950	1	1 (0.1)	1 (0.1)
Autosomal recessive polycystic kidney disease	731	43	43 (4.8)	43 (5.2)
Bardet-Biedl syndrome	110	4	4 (0.5)	4 (0.5)
Branchiootorenal syndrome	107	5	5 (0.6)	5 (0.6)
Caroli Disease	53,035	1	1 (0.1)	1 (0.1)
CHARGE syndrome	138	1	1 (0.1)	1 (0.1)
Congenital nephrotic syndrome	97,556	2	2 (0.2)	1 (0.1)
Congenital nephrotic syndrome type 4	656	6	6 (0.7)	6 (0.7)
Congenital nephrotic syndrome Finnish type	839	34	34 (3.8)	34 (4.1)
Congenital nephrotic syndrome type 2	656	17	17 (1.9)	17 (2.1)
Congenital nephrotic syndrome type 3	656	2	2 (0.2)	2 (0.2)
Cranioectodermal dysplasia	1515	1	1 (0.1)	1 (0.1)
Cystinosis	213	16	16 (1.8)	16 (1.9)
Dense deposit disease	93,571	1	1 (0.1)	
Denys-Drash syndrome	220	6	6 (0.7)	6 (0.7)
Ellis-Van Creveld syndrome	289	1	1 (0.1)	1 (0.1)
Fabry disease	324	1	1 (0.1)	1 (0.1)
Familial vesicoureteral reflux	289,365	12	12 (1.4)	12 (1.4)
Fanconi syndrome	3337	1	1 (0.1)	1 (0.1)
Fechtner syndrome	1984	1	1 (0.1)	1 (0.1)
Focal segmental glomerulosclerosis	656	72	72 (8.1)	72 (8.7)
Frasier syndrome	347	2	2 (0.2)	2 (0.2)
Glomerulonephritis	93,559	38	38 (4.3)	
Goodpasture disease	375	1	1 (0.1)	
Granulomatosis with polyangiitis	900	1	1 (0.1)	1 (0.1)
Hemolytic uremic syndrome	544,458	26	26 (2.9)	26 (3.1)
Henoch-Schonlein purpura	761	1	1 (0.1)	
Hepatorenal syndrome		3		
Hereditary endotheliopathy-retinopathy-nephropathy-stroke (HERNS) syndrome	63,261	1	1 (0.1)	1 (0.1)
Hypertensive nephrosclerosis		1		
Hypoplastic kidneys	93,101	10	10 (1.1)	
IgA nephropathy	761	8	8 (0.9)	
Jeune syndrome	474	1	1 (0.1)	1 (0.1)
Joubert syndrome with oculorenal anomalies	2318	13	13 (1.5)	13 (1.6)
Leopard syndrome	500	1	1 (0.1)	1 (0.1)
Lowe oculocerebrorenal syndrome	534	2	2 (0.2)	2 (0.2)
Lupus nephritis	300,345	8	8 (0.9)	
Megaureter syndrome with oculorenal anomalies	238,637	1	1 (0.1)	
Membranous nephropathy	97,560	3	3 (0.3)	
Methylmalonic acidemia	293,355	2	2 (0.2)	2 (0.2)
Microscopic polyangiitis	727	3	3 (0.3)	
Mitochondrial DNA-associated Leigh syndrome	255,210	2	2 (0.2)	2 (0.2)
Nail-patella syndrome	2614	1	1 (0.1)	1 (0.1)
Neonatal cortical necrosis		13		
Nephronophthisis	655	71	71 (8.0)	71 (8.6)
Nephropathy due to chemotherapy		1		
Neurogenic bladder	84,085	2	2 (0.2)	
Other nephropathy		31		
Primary hyperoxaluria type 1	93,598	14	14 (1.6)	14 (1.7)
Primary hyperoxaluria type 2	93,599	1	1 (0.1)	1 (0.1)
Prune belly syndrome	2970	9	9 (1.0)	9 (1.1)
Renal agenesis	411,709	7	7 (0.8)	
Renal coloboma syndrome	1475	3	3 (0.3)	3 (0.4)
Renal or urinary tract malformation	93,545	327	327 (36.9)	
Renal-hepatic-pancreatic dysplasia-Dandy-Walker cysts syndrome	3032	1	1 (0.1)	1 (0.1)
Schimke immune-osseous dysplasia	1830	1	1 (0.1)	1 (0.1)
Sirenomelia	3169	1	1 (0.1)	1 (0.1)
Split hand urinary anomalies spina bifida (Czeizel-Losonci syndrome)		2437	4	4 (0.5)
Townes-Brocks syndrome	857	1	1 (0.1)	1 (0.1)
Trauma		1		
VACTERL/VACTER association	887	2	2 (0.2)	2 (0.2)
Vasculitis	52,759	7	7 (0.8)	
Wilms tumor	654	2	2 (0.2)	

Tx: transplants

**Fig. 5:**
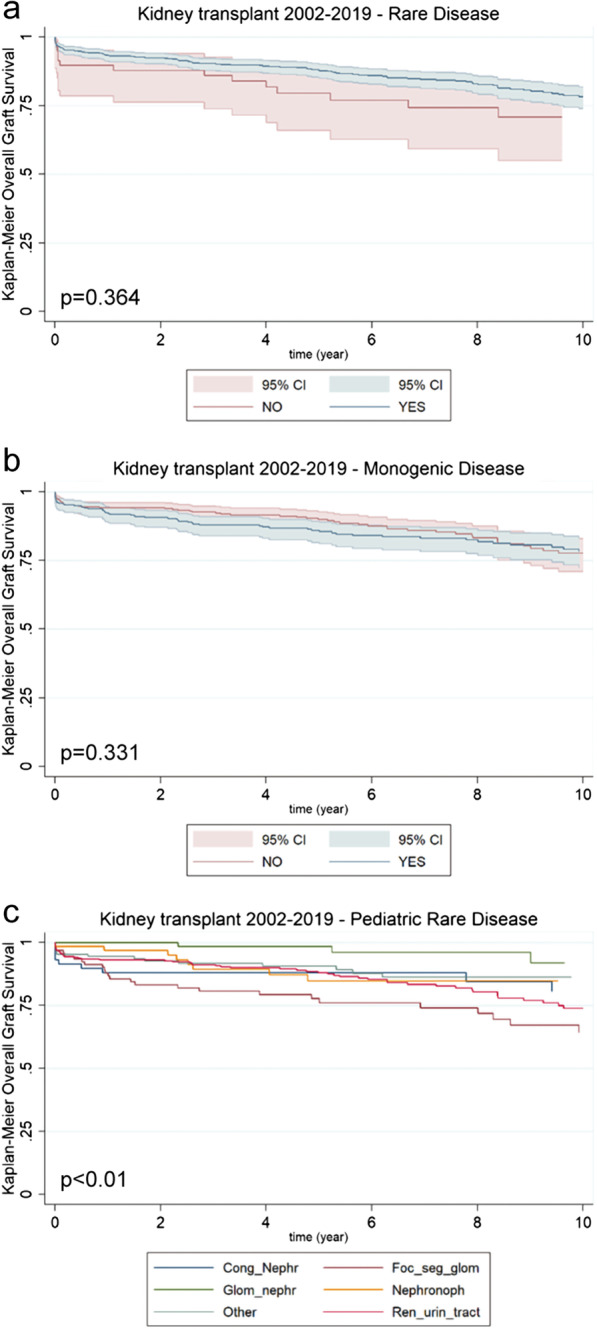
Survival of kidney transplanted patients in the 2002–2019 timeframe. **a** Kaplan–Meier curves comparing pediatric patients affected by rare diseases (green line; n = 758) vs. pediatric patients affected by non-rare diseases (red line; n = 59) [p = 0.364]. **b** Kaplan–Meier curves comparing pediatric patients affected by monogenic diseases (green line; n = 367) vs. pediatric patients affected by rare non-monogenic diseases (red line; n = 391) [p = 0.331]. **d** Kaplan–Meier curves comparing pediatric patients affected by rare diseases stratified on the basis of their diagnosis [*p* < 0.01]: congenital nephrosis (Cong_Nephr; blue line; n = 59); glomerular nephrosis (Glom_nephr; green line; n = 84); focal segmental glomerulosclerosis (Foc_seg_glom; red line; n = 95); nephronophtisis (Nephronoph; yellow line; n = 65); renal and urinary tract disorders (Ren_urin_tract; pink line; n = 326); other diseases (Other; purple line; n = 129)

In pediatric kidney transplants and similar to what observed for lung transplants, no significant differences were highlighted when comparing rare vs common diseases (p = 0.36; Fig. 5a) or when considering monogenic vs non-monogenic diseases (p = 0.33; Fig. 5b) at ten years after transplant. However, when plotting overall graft survival taking in consideration the most representative disease macro-categories, some significant differences appeared, with glomerular and congenital nephropathies showing the best outcome as opposed to renal and urinary tract abnormalities and focal segmental glomerulosclerosis (*p* < 0.01; Fig. 5c).

## Discussion

An ample body of literature comes out when surfing PubMed using “transplantation” or “organ transplants” as search keywords. Different aspects are addressed and discussed, mainly referring to clinical and surgical topics, as well as to immunological issues. In the last year, even the impact of COVID-19 has represented a “hot topic” in transplantation (22, 23). In this paper, solid organ transplants are addressed and investigated from a genetic perspective, which is a relatively unexplored field (17, 18, 24), thus strengthening the novelty of this work. We carried out a national survey, based on data included in the Italian Transplant Registry (TR), by focusing on disease causes that lead to organ failure thus finally requiring transplants. Specifically, we reported the Italian “state of the art”, considering both the adult and pediatric cohorts, with the aim of comparing these two subsets of patients, highlighting the percentage of undiagnosed patients included in the TR, and describing the frequency and distribution of rare and monogenic diseases in transplants recipients. Several points of discussion can be raised from this analysis.

The first observation that comes to light concerns the percentage of patients included in the TR without a disease diagnosis. Considering all transplants, a diagnosis is recorded in 40,909 (82.8%) resulting in a 17.8% of transplanted patients who remain undiagnosed, in line with previous data (25–27). When dividing by organs, heart and lung transplants present with the higher percentage of undiagnosed patients (n = 1,611, 33.1% and n = 583, 30.3%, respectively), followed by kidney transplants (n = 5,967, 23.5%), while only a minority of liver transplanted patients were without a diagnosis (n = 334, 1.9%). A possible explanation for the higher percentage of diagnosed patients in TR for liver transplants compared to the other organs may rely on the structure of the TR, allowing for liver, and only for this organ, three different levels of diagnostic information to be recorded.

The second observation regards the quote of diagnosed pediatric transplants (2,294 patients in total, 5.6% of all diagnosed transplanted patients). Depending on the organ considered, the percentage of diagnosed patients varies, being the highest for heart (8.5%), followed by liver (6.1%) and lung (7.0%). On the contrary, kidney presented the lower quote of diagnosed patients (4.5%). It is noteworthy that the frequencies and distribution of pathologies leading to organ transplants are different between the adult and pediatric cohorts, especially when analysing kidney and liver transplants. Moreover, the quote of pediatric kidney transplants is lower compared to the other organs, when taking in consideration the overall number of transplants for each organ. This lower percentage is probably linked to different factors: firstly, kidney diseases may take a longer period of time to lead to organ failure. In line with this hypothesis, the mean age of enrollment in the TR is higher for kidney patients (9.86 years) compared to heart and liver ones (7.84 and 4.06 years, respectively). Secondly, kidney transplant does not represent a lifesaving treatment as other options can temporarily supply to organ dysfunction.

The third point of discussion is centred on the disease categories within the pediatric cohort. Overall, we listed 128 different diseases, with kidney transplants contributing the most to this catalogue (51.5%), followed by liver (30.5%), heart (10.9%) and lung (7.0%). However, it must be noted that the number of pediatric patients with diagnosis who underwent either a heart or lung transplant is relatively small. Interestingly, out of 128 disease categories identified, 117 are included in the main catalogues of rare diseases, meaning that 92.7% of the pediatric cohort is affected by this type of diseases. This proportion varies slightly depending on the organ involved, being higher for thoracic organ transplants (98.9%) compared to abdominal ones (94.7% for kidney and 88.7% for liver). An interesting result that has been highlighted by this survey is that a considerable percentage (65.9%) of the pediatric patients presenting with a rare disease is affected by a monogenic condition. As expected, lung, heart and liver transplants presented the higher quote of monogenic diseases (88%, 81.7% and 74.6%, respectively) at variance with kidney transplants, where < 50% of patients are affected by a disease of monogenic origin. This significant difference can be explained by the fact that within kidney, some categories of rare diseases may include a quote of monogenic pathologies (e.g., CAKUT, glomerulonephritis) thus leading to an underestimation of the real percentage. The distribution of affected patients seems to be independent of the sex in heart and liver transplants, while a clear association was present for lung and kidney ones. In the first case, there was a prevalence (63.8%) of female patients likely reflecting the fact that the great majority of the cohort was affected by cystic fibrosis. Several papers in literature suggest that a sex dichotomy exists for this disease, despite improved therapies, with female patients experiencing a slightly worse prognosis, both in terms of mortality and susceptibility to chronic infections (28–31). On the contrary, for kidney transplants, there was a prevalence of male subjects (61.8%). In part, these data can be explained by the fact that some diseases are characterized by an X-linked mode of inheritance (e.g., Alport syndrome) thus affecting male subjects. Beside the genetic aspect, these data are in line with evidence reported in literature showing that worldwide there is a milder male predominance among kidney transplant patients (32, 33).

When considering the graft survival, as expected (34), a clear statistically significant difference can be highlighted comparing the pediatric and adult cohorts, with the former group showing the best survival at ten years after transplants. The only exception, but in line with reported data (20, 35), is represented by lung, probably in part as a consequence of the over-representation of cystic fibrosis both in children and adults. Within the pediatric cohort, a diagnosis of rare or monogenic disease is not by itself sufficient to predict graft survival probability. Indeed, a clear and unique trend was not evident by this analysis.

Overall, this survey has drawn attention on the significant proportion of patients included in a waiting list or already transplanted who lack a disease diagnosis at the time of transplant (25–27). Moreover, a prominent quote of patients requiring organ transplantation are affected by rare and monogenic diseases (17, 18). These data open the discussion on the possibility to improve the diagnostic power by introducing genetic testing as part of the clinical flow before organ transplants, at least for some patient categories (36–39). The availability of sequencing platforms, the possibility to design selective gene panels for the analysis as well as the reduced costs of genetic tests may be in favour of this hypothesis, while avoiding doubtful exams and diagnosis. Having the correct diagnosis of the original disease that leads to organ insufficiency is relevant for many reasons, including i) the identification of the best therapeutic window to perform transplantation, ii) prevention of post-transplant complications that can be related to the original disease, iii) adoption of specific therapeutic regimens. This issue becomes even more relevant for rare and monogenic diseases where the genetic testing is crucial for a univocal diagnosis.

A further point to be stressed is that, at least for a small proportion of these patients, novel therapeutic approaches may represent a viable alternative to transplantation. While this is currently available mostly in the context of clinical trials, it is conceivable that in the near future targeted treatments, such as gene therapy/editing and stem cell-based therapies may become available for an increasingly higher number of rare diseases (40–42).

Lastly, a final consideration regards the economic impact of transplants as a treatment strategy compared to conventional treatments. At least in Western countries, it is well known that transplantation is cost-effective compared to other options (43, 44). As an example, for kidney, transplantation is the best choice compared to dialysis, with an initial cost of approximately 45,000 Euros that drops-off to 8,000 Euros starting from the second year after transplantation.

## Conclusions

**Fig. 6:**
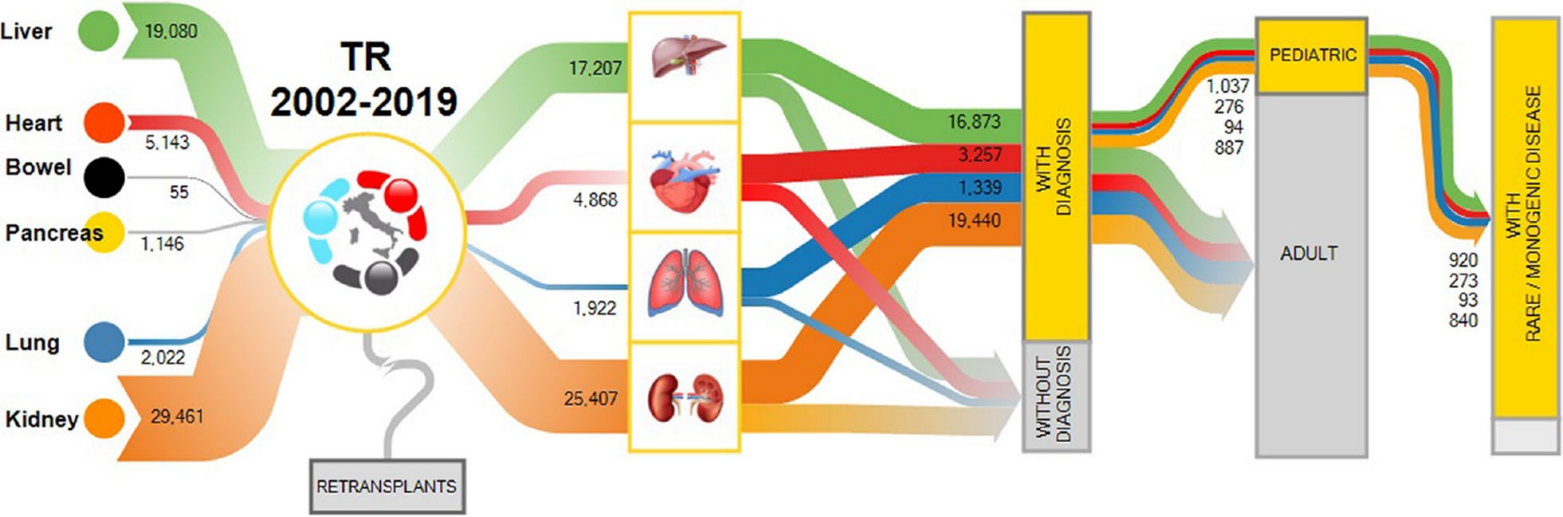
Sankey diagram showing the analytical flow adopted in the study. The diagram showed the different parameters that were taking into account in the present analyses: transplanted patients registered in the transplanted register (TR) in the 2002–2019 timeframe, cohort selection and inclusion/exclusion criteria, classification on the basis of presence/absence of a diagnosis indicated in the TR, analysis of the pediatric cohort based on a diagnosis of rare and/or monogenic diseases. Single pancreas and bowel transplants were excluded from the analysis, as well as re-transplanted patients

**Fig. 7:**
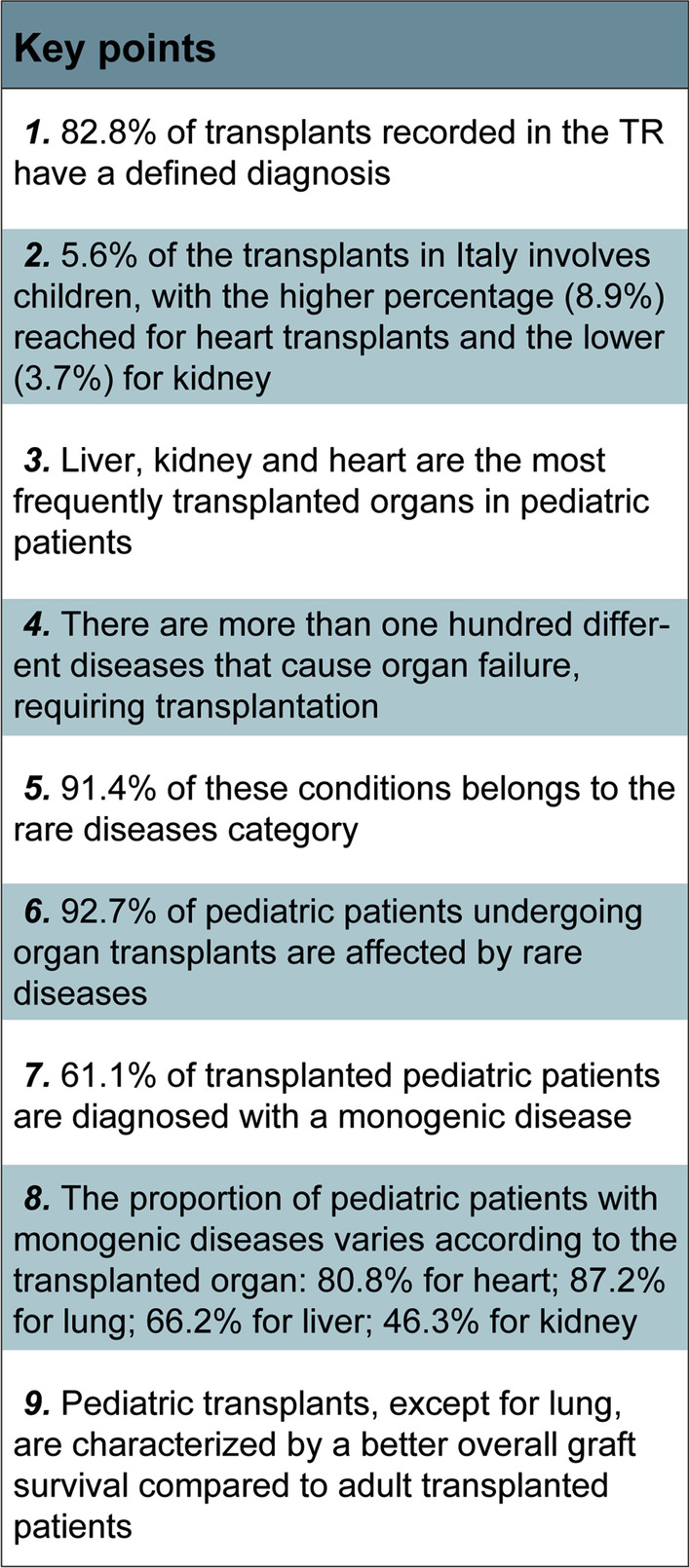
Key points of the study. Highlights of the study emerging from this national survey of patients included in National Transplants Registry

This work was designed to provide an updated snapshot of organ transplants in Italy, considered from a genetic perspective. We went through the Italian Transplant Registry, considering both the adult and pediatric cohorts, focusing on disease diagnoses that lead to organ failure finally requiring transplants (Fig. 6). Remarkably, in 1 patient out of 5, transplantation was performed in patients lacking a diagnosis. Within the diagnosed subset, the great majority of the pediatric patients presented with rare genetic diseases, most of them being affected by monogenic pathologies (Fig. 7).

The results of this work represent, to our knowledge, the first national survey on genetic diseases leading to organ transplants, being a novelty in the field. Moreover, they represent a starting point for future considerations on the relevance of disease diagnosis for patient management, taking into account also the increasing spread of sequencing technologies that allow for a clear genetic diagnosis.

## Methods

### Italian transplant registry

The Transplant Registry (TR) is entrusted to the Transplant Information System (TIS), which is an infrastructure for the management of data related to the activity of the National Transplant Network, established and regulated by Italian Laws (no. 91/99 and Decree of the Ministry of Health n. 130 of 20 August 2019).

### Clinical data and rare and monogenic disease diagnosis

Several data are collected by the TR, including patients’ demographic and clinical information, survival after transplant, and indication of the disease that caused end-stage organ failure. To this purpose, a specific in series classification is used for each organ to define macro- and sub-categories of pathologies, the latter used to provide more detailed features of the diagnosis.

To identify rare and monogenic diseases in our cohort, a list of these pathologies was generated, matching entries from the National Organization for Rare Disorders (NORD; https://rarediseases.org/), Orphanet database (https://www.orpha.net/), the Genetic and Rare diseases information center (NIH-GARD, https://rarediseases.info.nih.gov/) and Online Mendelian Inheritance in Man catalog (OMIM, https://www.omim.org/). In the case of the kidney, the available diagnosis in the TR did not allow for an unambiguous classification, and the pediatric kidney transplant centers were asked to fill in a specific form in order to identify the diagnosis of the disease in a more precise manner, allowing for rare and monogenic disease classification.

### Statistical analyses

The Kaplan–Meier method was used to analyze overall graft survival (death non censored graft survival). These analyses were carried out considering transplant recipients i) with a follow up and ii) that underwent a first transplant. The corresponding number of patients considered are indicated in the figure legends. The log rank test was used to evaluate the statistical significance. For the age of registration in the transplant list and the age at transplant for the pediatric cohort, values were expressed as median (interquartile range) unless otherwise specified. The Shapiro–Wilk normality test was used to evaluate normality distribution for continuous or ordinal dependent variable. Kruskal–Wallis test was used to assess for significant differences on a continuous dependent variable. P values < 0.05 were considered statistically significant. All data were analyzed using STATA (v 16.1 Copyright 1985–2019 StataCorp LP 4905 Lakeway Drive College Station, Texas 77,845 USA).

## Supplementary Information

**Additional file 1.** Additionale file 1 includes Supplementary Table 1 showing combined transplants in the pediatricand adult cohorts included in the study and a Supplementary Table 2 listing Transplant Centers that enrolledpediatric and adult patients included in the study and present in the Transplant Registry.

## Data Availability

The datasets generated and/or analysed during the current study are not publicly available since they are part of the National Transplantation Center only, but are available from the corresponding author on reasonable request.
