# Supplementary material for: The frequency of rare and monogenic diseases in pediatric organ transplant recipients in Italy
Source: Orphanet J Rare Dis. 2021 Sep 4;16:374. doi: 10.1186/s13023-021-02013-x (PMC8418291; doi:10.1186/s13023-021-02013-x)
Supplement: Supplementary file 1 — Additional file 1. Additionale file 1 includes Supplementary Table 1 showing combined transplants in the pediatricand adult cohorts included in the study and a Supplementary Table 2 listing Transplant Centers that enrolledpediatric and adult patients included in the study and present in the Transplant Registry. [file 13023_2021_2013_MOESM1_ESM.docx]

| **Supplementary Table 1.** Combined transplants in the pediatric and adult cohorts included in the study. | | | |
| --- | --- | --- | --- |
| **Transplanted organ** | **Pediatrics (n. 171)** | **Adults (n. 4,201)** | **Total (n. 4,372)** |
| Heart-liver |  | 12 | 12 |
| Heart-pancreas |  | 1 | 1 |
| Heart-lung (bi-pulmonary) | 7 | 18 | 25 |
| Liver-bowel | 2 |  | 2 |
| Liver-pancreas | 3 | 13 | 16 |
| Liver-pancreas-bowel | 4 | 6 | 10 |
| Liver-pancreas- lung (bi-pulmonary) | 1 | 1 | 2 |
| Liver-lung (bi-pulmonary) | 2 | 7 | 9 |
| Lung (bi-pulmonary) | 106 | 1,266 | 1,372 |
| Kidney-heart | 4 | 39 | 43 |
| Kidney-heart-liver |  | 1 | 1 |
| Kidney-liver | 35 | 302 | 337 |
| Kidney-liver-pancreas |  | 1 | 1 |
| Kidney-pancreas | 1 | 741 | 742 |
| Kidney-lung |  | 1 | 1 |
| Kidney (bi-renal) | 5 | 1,787 | 1,792 |
| Kidney (bi-renal)-liver | 1 | 6 | 7 |

|  |  | | |  | | |  | | |  | | |  |  |  |
| --- | --- | --- | --- | --- | --- | --- | --- | --- | --- | --- | --- | --- | --- | --- | --- |
| **Supplementary Table 2.** Transplant Centers that enrolled pediatric and adult patients included in the study and present in the Transplant Registry. | | | | | | | | | | | | | | | |
|  | **Kidney** | | | **Liver** | | | **Heart** | | | **Lung** | | | **Total** | | **Overall Number of patients** |
| **Transplant Center** | **Adu** | **Ped** | **Tot** | **Adu** | **Ped** | **Tot** | **Adu** | **Ped** | **Tot** | **Adu** | **Ped** | **Tot** | **Adu** | **Ped** |  |
| A.O. MONALDI |  |  | 0 |  |  | 0 | 331 | 21 | 352 |  |  | 0 | **331** | **21** | **352** |
| A.O. SAN CAMILLO - FORLANINI | 368 |  | 368 | 434 |  | 434 | 206 |  | 206 |  |  | 0 | **1,008** | **0** | **1,008** |
| A.O. UNIVERSITARIA DI CATANIA | 519 | 4 | 523 |  |  | 0 |  |  | 0 |  |  | 0 | **519** | **4** | **523** |
| A.O.U. CAREGGI - FIRENZE | 638 | 3 | 641 |  |  | 0 |  |  | 0 |  |  | 0 | **638** | **3** | **641** |
| A.O.U. PISANA | 638 | 1 | 639 | 1,871 | 8 | 1,879 |  |  | 0 |  |  | 0 | **2,509** | **9** | **2,518** |
| A.O.U. S. MARTINO - GENOVA | 719 | 165 | 884 | 440 | 2 | 442 | 244 | 1 | 245 |  |  | 0 | **1,403** | **168** | **1,571** |
| A.O.U. SENESE - S.M. alle SCOTTE | 772 | 1 | 773 |  |  | 0 |  |  | 0 | 160 | 2 | 162 | **932** | **3** | **935** |
| A.O. NIGUARDA CA' GRANDA - MILANO | 883 | 1 | 884 | 1,145 |  | 1,145 | 497 | 32 | 529 | 57 | 1 | 58 | **2,582** | **34** | **2,616** |
| A.O.U. Città della Salute e della Scienza, Molinette Hospital | 2,487 | 88 | 2,575 | 2,139 | 141 | 2,280 | 345 | 51 | 396 | 255 | 19 | 274 | **5,226** | **299** | **5,525** |
| A.O.U. CONSORZIALE POLICLINICO di BARI | 874 | 27 | 901 | 330 |  | 330 | 76 |  | 76 |  |  | 0 | **1,280** | **27** | **1,307** |
| A.O.U. MAGGIORE DELLA CARITA' - NOVARA | 179 |  | 179 |  |  | 0 |  |  | 0 |  |  | 0 | **179** | **0** | **179** |
| A.O. V. EMANUELE FERRAROTTO S. BAMBINO |  |  | 0 |  |  | 0 | 36 |  | 36 |  |  | 0 | **36** | **0** | **36** |
| A.O.U. POLICLINICO TOR VERGATA | 658 | 1 | 659 | 431 | 3 | 434 |  |  | 0 |  |  | 0 | **1,089** | **4** | **1,093** |
| A.O. "VITO FAZZI " | 1 |  | 1 |  |  | 0 |  |  | 0 |  |  | 0 | **1** | **0** | **1** |
| A.O "A. CARDARELLI" |  |  | 0 | 635 | 2 | 637 |  |  | 0 |  |  | 0 | **635** | **2** | **637** |
| A.O. DI PADOVA | 1,305 | 201 | 1,506 | 1,221 | 112 | 1,333 | 409 | 35 | 444 | 361 | 26 | 387 | **3,296** | **374** | **3,670** |
| A.O DI PERUGIA | 274 |  | 274 |  |  | 0 |  |  | 0 |  |  | 0 | **274** | **0** | **274** |
| A.O G. BROTZU | 550 | 4 | 554 | 322 |  | 322 | 97 | 5 | 102 |  |  | 0 | **969** | **9** | **978** |
| A.O. POLICLINICO - MODENA | 509 | 2 | 511 | 725 | 1 | 726 |  |  | 0 |  |  | 0 | **1,234** | **3** | **1,237** |
| A.O. S. M. MISERICORDIA UDINE | 646 | 1 | 647 | 469 |  | 469 | 423 | 3 | 426 |  |  | 0 | **1,538** | **4** | **1,542** |
| A.O.U. POLICLINICO | 45 | 1 | 46 |  |  | 0 |  |  | 0 |  |  | 0 | **45** | **1** | **46** |
| IRCCS S. RAFFAELE - MILANO | 558 |  | 558 |  |  | 0 |  |  | 0 |  |  | 0 | **558** | **0** | **558** |
| Is.Me.T.T. | 325 | 20 | 345 | 795 | 134 | 929 | 149 | 2 | 151 | 137 | 12 | 149 | **1,406** | **168** | **1,574** |
| OORR S. GIOVANNI DI DIO E RUGGI D`ARAGONA | 291 | 2 | 293 |  |  | 0 |  |  | 0 |  |  | 0 | **291** | **2** | **293** |
| OSPEDALE BIANCHI - MELACRINO - MORELLI | 246 | 1 | 247 |  |  | 0 |  |  | 0 |  |  | 0 | **246** | **1** | **247** |
| OSPEDALE CA' FONCELLO - TREVISO | 599 | 1 | 600 |  |  | 0 |  |  | 0 |  |  | 0 | **599** | **1** | **600** |
| IST. NAZ.LE PER CURA TUMORI - MILANO |  |  | 0 | 543 | 5 | 548 |  |  | 0 |  |  | 0 | **543** | **5** | **548** |
| OSP. G. PASQUINUCCI (OSP. PEDIATRICO APUANO) - MASSA |  |  | 0 |  |  | 0 |  | 1 | 1 |  |  | 0 | **0** | **1** | **1** |
| OSP. CIVILE MAGGIORE - VERONA | 1,061 | 3 | 1,064 | 485 |  | 485 | 279 | 3 | 282 | 2 |  | 2 | **1,827** | **6** | **1,833** |
| OSP. CIVILE S. SALVATORE - L'AQUILA | 500 |  | 500 |  |  | 0 |  |  | 0 |  |  | 0 | **500** | **0** | **500** |
| OSP. DI CIRCOLO E FONDAZIONE MACCHI | 652 |  | 652 |  |  | 0 |  |  | 0 |  |  | 0 | **652** | **0** | **652** |
| OSP. DI VICENZA | 396 |  | 396 |  |  | 0 |  |  | 0 |  |  | 0 | **396** | **0** | **396** |
| OSP. MAGGIORE - PARMA | 763 | 5 | 768 |  |  | 0 |  |  | 0 |  |  | 0 | **763** | **5** | **768** |
| OSP. MAGGIORE POLICLINICO - MILANO | 630 | 130 | 760 | 708 | 15 | 723 |  |  | 0 | 247 | 12 | 259 | **1,585** | **157** | **1,742** |
| OSP. PAPA GIOVANNI XXIII - BERGAMO | 629 | 13 | 642 | 856 | 472 | 1328 | 359 | 75 | 434 | 130 | 23 | 153 | **1,974** | **583** | **2,557** |
| OSP. PEDIATRICO BAMBINO GESU' - ROMA | 2 | 217 | 219 | 2 | 164 | 166 | 24 | 137 | 161 | 5 | 16 | 21 | **33** | **534** | **567** |
| OSP. POLICLINICO S. MATTEO - PAVIA | 433 |  | 433 |  |  | 0 | 474 | 11 | 485 | 221 | 1 | 222 | **1,128** | **12** | **1,140** |
| OSP. SAN CAMILLO DE' LELLIS |  |  | 0 |  |  | 0 | 29 |  | 29 |  |  | 0 | **29** | **0** | **29** |
| OSP. S.S. ANNUNZIATA -SASSARI | 115 | 1 | 116 |  |  | 0 |  |  | 0 |  |  | 0 | **115** | **1** | **116** |
| P.O. CIVICO E BENFRATELLI | 671 | 2 | 673 |  |  | 0 | 1 | 9 | 10 |  |  | 0 | **672** | **11** | **683** |
| POLICLINICO UMBERTO I - ROMA | 624 | 12 | 636 | 394 | 9 | 403 | 1 |  | 1 | 151 | 10 | 161 | **1,170** | **31** | **1,201** |
| POLICLINICO UNIVERSITARIO A. GEMELLI - ROMA | 596 |  | 596 | 415 | 2 | 417 |  |  | 0 |  |  | 0 | **1,011** | **2** | **1,013** |
| P.O. SPEDALI CIVILI BRESCIA | 770 | 3 | 773 |  |  | 0 |  |  | 0 |  |  | 0 | **770** | **3** | **773** |
| P.O. ANNUNZIATA | 172 |  | 172 |  |  | 0 |  |  | 0 |  |  | 0 | **172** | **0** | **172** |
| P.O. UMBERTO I - ANCONA | 487 | 2 | 489 | 525 |  | 525 |  |  | 0 |  |  | 0 | **1,012** | **2** | **1,014** |
| S. ORSOLA-MALPIGHI - BOLOGNA | 1,192 | 14 | 1,206 | 1,245 | 7 | 1,252 | 456 | 46 | 502 | 74 |  | 74 | **2,967** | **67** | **3,034** |
| UNIV. DI NAPOLI - FEDERICO II – MEDICAL DEPARTMENT | 702 | 2 | 704 |  |  | 0 |  |  | 0 |  |  | 0 | **702** | **2** | **704** |
| **Total** | **24,479** | **928** | **25,407** | **16,130** | **1,077** | **17,207** | **4,436** | **432** | **4,868** | **1,800** | **122** | **1,922** | **46,845** | **2,559** | **49,404** |
| Adu: adults; Ped: pediatrics; Tot: total; A.O.: Azienda Ospedaliera (Hospital); A.O.U.: Azienda Ospedaliera Universitaria (University Hospital); IRCCS: Istituto di Ricovero e Cura a Carattere Scientifico; Is.Me.T.T.: Istituto Mediterraneo per i Trapianti; P.O.: Presidio ospedaliero (Hospital); OSP.: Ospedale (Hospital); UNIV.: University | | | | | | | | | | | | | | | |
